# Dairy consumption and vitamin D concentration in adolescents with challenge-confirmed cow’s milk allergy during infancy

**DOI:** 10.1038/s41430-024-01477-x

**Published:** 2024-07-28

**Authors:** Sonja Piippo, Helena Hauta-alus, Mirva Viljanen, Erkki Savilahti, Mikael Kuitunen

**Affiliations:** 1 Pediatric Research Center, University of Helsinki and Helsinki University Hospital, Children’s Hospital, Helsinki, Finland; 2 Faculty of Medicine, Research Program for Clinical and Molecular Metabolism (CAMM), University of Helsinki, Helsinki, Finland; 3 Population Health Unit, National Institute for Health and Welfare (THL), Public Health Research team, Helsinki, Finland; 4 PEDEGO Research Unit, MRC Oulu, Oulu University Hospital and University of Oulu, Oulu, Finland

**Keywords:** Paediatrics, Immunological disorders, Nutrition

## Abstract

**Background/Objectives:**

Milk is an important source of dietary calcium and, if fortified, vitamin D. Cow’s milk allergy (CMA) is treated with a milk elimination diet. Although most children become tolerant by age 3 years, some continue dairy avoidance. It remains unclear whether adolescents with a history of CMA adopt similar milk consumption as their peers. We assessed dairy consumption and concentration of serum 25-hydroxyvitamin D (25(OH)D) in adolescents with either confirmed CMA or a negative CMA challenge in infancy (CMA-refuted group) and age-matched controls.

**Subjects/Methods:**

This study is based on a previously reported randomized controlled trial from 1999 to 2002 on the treatment effect of probiotics on atopic eczema in participants aged <12 months (*n* = 230) with data on CMA status. We followed up these participants, aged 15–18 years, in 2017 (*n* = 104). A 20-item food frequency questionnaire assessed dairy consumption. An automated immunoassay measured 25(OH)D concentration.

**Results:**

Median dairy product consumption did not differ between adolescents with CMA (449 g/d, *n* = 40), the CMA-refuted group (566 g/d, *n* = 36), and controls (235 g/d, *n* = 51) (*P* = 0.117). Median 25(OH)D concentrations were 76.0, 79.3, and 80.8 nmol/l, respectively (*P* = 0.844). Among participants, 93% were vitamin D sufficient (25(OH)D ≥ 50 nmol/l), with no differences between groups (*P* = 0.914).

**Conclusion:**

Among adolescents with a history of CMA during infancy, our study found no reintroduction failure of milk and no difference in vitamin D insufficiency rate compared with peers. Current management of CMA seems to adequately minimize later nutritional disadvantages associated with a cow’s milk elimination diet.

## Introduction

Cow’s milk allergy (CMA) is diagnosed in 0.5–2.4% of European children [1, 2] and is treated with a milk-elimination diet. Milk contains important nutrients such as protein, calcium, and iodine and is in many countries fortified with cholecalciferol (vitamin D_3_). In Finland, this fortification, recommended by the National Nutrition Council, is for liquid dairy products (1 µg/100 ml) and for fat spreads (20 µg/100 g) [3]. While this fortification is not mandatory, it is implemented in practically all milk products. In Finnish children, milk consumption is the major determinant of serum 25-hydroxyvitamin D (25(OH)D) concentration [4] and the main source of calcium. Children on a milk-elimination diet are at risk of nutritional inadequacies, stunted growth, and reduced bone mineral density [5, 6]. In Finnish children, a history of CMA has been associated with a lower vitamin D concentration [7]. Most children, over time, spontaneously become tolerant. In a Danish cohort study, at the age of 3 years, 76% of patients with IgE-mediated CMA, and 100% of patients with non-IgE-mediated CMA, had achieved tolerance. At the age of 15 as well as 26 years, 5% (*n* = 1) remained allergic [8]. A Finnish study reported persistent CMA among 15% of children at the age of 8.6 years, all of them IgE-mediated [9]. Even tolerant children with a history of an elimination diet (milk, egg, wheat), compared with those without any history of food elimination, have shown, at school age, a reduced height standard deviation score (SDS) [10].

Food preferences are largely formed by the age of 3 years [11]. Children adhering to a milk-elimination diet have shown restricted overall diet variability, which may negatively affect nutritional intake and long-term eating behavior [12, 13]. A Swedish study observed reintroduction failure among 2 out of 8 participants at an 18-month follow-up visit among previously allergic children who had proved, by double-blind, placebo-controlled, oral food challenge (DBPCFC), milk tolerant at age 11–12 years [14].

There is scarce data on dairy product consumption by adolescents who have become cow’s milk tolerant after treatment with a milk elimination diet during infancy nor adolescents with a negative CMA DBPCFC during infancy. Also, in these adolescents, 25(OH)D concentration and dietary intake of vitamin D remain unclear. Adolescents represent a demographic that has received limited research attention. Dietary habits frequently undergo transitional changes during this developmental stage.

We hypothesized that adolescents with a history of CMA during infancy who are now tolerant, compared with nonallergic peers, consume fewer dairy products and that these children have a lower 25(OH)D concentration. We also examined whether a history of CMA was associated, in adolescence, with differences in height or diet quality as measured by the Healthy Eating Index (HEI) or a prevalence of special diets.

## Materials & methods

### Design and participants

This study is based on a previously reported randomized controlled trial from 1999 to 2002, in which all participants had established CMA status by DBPCFC. Recruitment to the original study was among children who, at the mean age of 6.4 months, had been referred to a tertiary allergy hospital in Helsinki on suspicion of CMA based on atopic eczema. Among the original 230 children, 120 had positive DBPCFCs and were diagnosed with CMA. This study protocol has previously been reported in detail [15]. Briefly, the original study examined the effect of probiotics on the symptoms of atopic eczema during a 4-week placebo-controlled intervention. In 2017, an invitation to participate in our follow-up study was sent to all participants from this trial. Age-matched controls were recruited via collaboration with nurses in school health care in Helsinki and advertising on social media. Participants were asked to fill out two study questionnaires and attend a study visit.

**Fig. 1:**
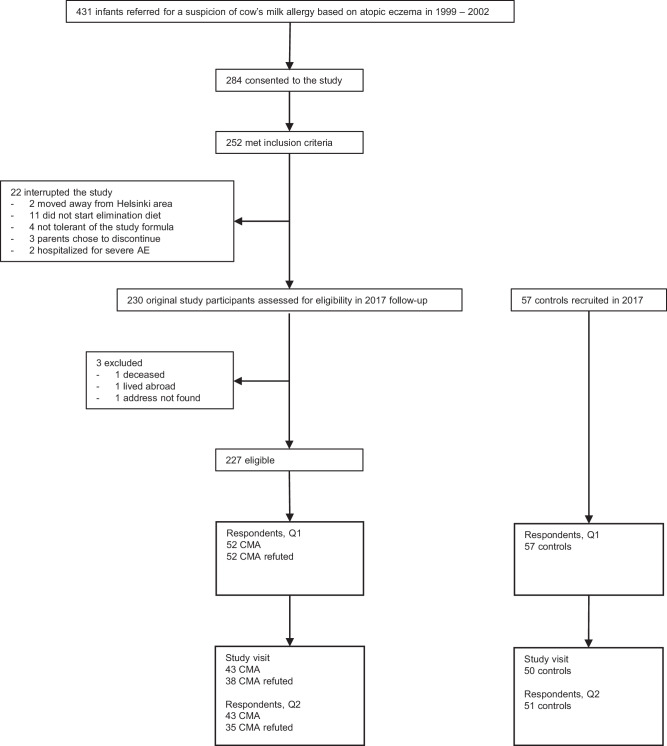
Flowchart of the study. AE atopic eczema, Q1 questionnaire one, Q2 questionnaire two, CMA cow’s milk allergy—positive, double-blind, placebo-controlled, oral cow’s milk challenge during the original study; CMA refuted: negative, double-blind, placebo-controlled, oral cow’s milk challenge during the original study. Inclusion criteria for the original study were: 1. age <12 months at the beginning of the study; 2. symptoms suggestive of CMA, the required symptom being AE; 3. no regular use of probiotic preparations (longer than 1 week within 6 weeks prior to the beginning of the study).

Out of 230 original study participants, three were non-contactable. Among the 227 original participants contacted, 104 (46%) consented to this follow-up study. The food frequency questionnaires (FFQs) were filled in by 79 participants (35%). Among them, during infancy, 43 had CMA and 36 had a negative CMA challenge (CMA refuted). Among 57 recruited controls, 51 (89%) filled in the FFQs (Fig. 1).

The power calculation was based on dairy consumption comparison between two groups. Group size of 25 was calculated to give 0.8 power with an *α* error of 0.05 based on a mean consumption of 455 g/d (SD = 228 g/d) among 2 year old healthy children [16] while the mean consumption of dairy products in previously cow’s milk allergic children 6 months–3 years of age at the first visit after tolerance achievement was 270 g/d (Jetta Tuokkola, unpublished data, personal communication).

### Dietary assessment

Two FFQs were distributed in electronic or paper format, as per participant preference. One FFQ collected data on dietary sources of vitamin D. This FFQ is not validated but has previously been used in several studies to estimate dietary vitamin D intake from food [17, 18]. It assessed, based on prespecified standard portion sizes, the quantity of milk that was (1) drunk, (2) added to coffee or tea, (3) consumed with cereal or muesli, and (4) used in preparing porridge, and yogurt. It also assessed other dietary sources of vitamin D, including eggs, food containing eggs (e.g., pancakes), fish, poultry, meat other than poultry, wild mushrooms, fat spread, and cooking fat. For each food item participants evaluated their consumption frequency on a daily, weekly, or monthly basis, as appropriate. The consumption frequencies of liquid dairy food items were converted into daily frequencies and then added up to derive the overall consumption of liquid dairy products. Participants specified the name of the milk, yogurt, fat spread, and cooking fat they most often consumed and what fish they had consumed during the past month. The Fineli database, a national food composition database managed by the Finnish Institute for Health and Welfare, and nutrition fact tables from food package labels were utilized to calculate vitamin D intake from the food.

Another, 22-item, semi-quantitative, FFQ on dietary patterns was applied to collect data on cheese consumption and to calculate a HEI comparable to the HEI-2005 [19] based on the Nordic Nutrition Recommendations 2012 [20]. The maximum HEI score, indicating the healthiest diet, was 50 points. This questionnaire is not validated but was developed for a study on associations between lifestyle factors and bone characteristics in subjects with early-onset severe obesity [21]. A portion size of cheese was defined as one slice or 10 g. Participants chose a matching consumption frequency (not at all, once a month or less often, 2–3 times a month, once a week or 4 times a month, 2–3 times a week, once a day, 2–3 times a day, 4–6 times a day or more frequently). Based on these responses, we calculated daily cheese intake. The calculation of total dairy product consumption combined liquid dairy from one FFQ with cheese consumption from the other FFQ.

Participants filled out the FFQs based on the past month’s recollection. Not all sources of dairy foods were represented in the FFQs and therefore we did not measure total milk intake. Data on special diets (vegetarian with consumption of fish/eggs/dairy, vegan, gluten-free, low-lactose, lactose-free, organic milk only, other) and dietary supplements was collected. This data revealed that one participant continuously had symptomatic CMA and they were thus excluded from the milk and liquid dairy consumption analyses.

### Anthropometric variables and biochemical markers

The research physician (SP) examined participants during a study visit. A research nurse measured weight (kg) and height (cm). To adjust for age and sex, height was converted into SDS and weight into body mass index-for-age (BMI-for-age), based on Finnish references [22]. The original study collected data on height SDS and length-adjusted weight percentage at the start of the DBPCFC. A venous blood sample was drawn after overnight fasting. The concentration of serum 25(OH)D was analyzed with a fully automated immunoassay (IDS-iSYS, Immunodiagnostic Systems Ltd, Boldon, UK) which has been assessed reliable in monitoring vitamin D therapy [23]. This assay uses acridinium-labeled sheep polyclonal anti-25(OH)D antibodies induced to chemiluminescence (vendor: Immunodiagnostic Systems Ltd Catalog number: IS-2700S). [24] The intra-assay coefficient of this method variation was less than 5% [25]. Adherence to the Vitamin D External Quality Assessment Scheme ensured reproducibility. The bias against NIST standard was ≤10% during year 2017. This concentration was measured in May–June in 17 participants and in August–November in 114 participants. The accredited Central Laboratory of Helsinki University Hospital (HUSLAB), with standard methodology, analyzed plasma calcium and inorganic phosphate.

### Statistical analysis

Visual inspection of histograms and the Kolmogorov–Smirnov test estimated the normality of the variable distributions. Based on the distribution normality, continuous variables were analyzed with ANOVA or the Kruskal–Wallis test. Continuous variables in two categories were analyzed by the Mann–Whitney *U* test. BMI-for-age was log-transformed into a normal distribution. Categorical variables were compared by the chi-squared test or Fisher’s exact test, as appropriate. The statistical tests were two-sided. Levene’s test was applied to assess equality of variance for parametric tests. To minimize the risk of a Type 1 error, pairwise comparisons were conducted post-hoc. Statistical significance was set at *P* < 0.05. Statistical analyses were conducted with SPSS Statistics for Windows, version 25 (IBM Corp., Armonk, NY, USA).

## Results

### Participant characteristics

Between groups, demographic characteristics were similar except for sex, as 84% of the control group was female vs 49% and 50% in the CMA and CMA-refuted groups, respectively (Table 1). Observations from the original study on mean height SDS in the CMA (–0.22) and CMA-refuted groups (–0.21) did not differ (*P* = 0.40), nor did median length–adjusted weight percentage in the CMA (–2.0%) and CMA-refuted groups (–2.7%) (*P* = 0.68). Neither were there differences in height SDS or BMI-for-age in this follow-up study. As previously reported, loss to follow-up among the original participants was more common among patients who, during the original study, had a mother that smoked (*P* = 0.024), and among males (*P* = 0.004) [26].

**Table 1:** Participant characteristics of the total sample and the three study groups.

	All (*n* = 130)	Cow’s milk allergy (*n* = 43)	Cow’s milk allergy refuted (*n* = 36)	Controls (*n* = 51)
Age—years	17.2 (0.6)	17.2 (0.4)	17.2 (0.4)	17.2 (0.8)
Sex—female	82 (63.1)	21 (48.8)	18 (50)	43 (84.3)^d^
Ethnicity—northern European^a^	111 (94.9)	38 (100)	31 (96.9)	42 (89.4)
Height—standard deviation score^a^	0.10 (1.23)	0.02 (1.23)	−0.20 (1.29)	0.37 (1.17)
Body mass index-for-age—median, (Q1, Q3)^a^	21.7 (20.0, 23.8)	22.2 (20.5, 24.3)	22.0 (19.5, 29.1)	21.0 (19.8, 23.1)
Siblings	118 (90.8)	38 (88.4)	33 (91.7)	47 (92.2)
Maternal age at birth—years^b^	30.8 (5.0)	30.6 (4.4)	31.5 (4.5)	30.4 (5.7)
Maternal smoking (ever), yes	45 (34.6)	15 (34.9)	15 (41.7)	15 (29.4)
Paternal smoking (ever), yes	50 (38.5)	13 (30.2)	15 (41.7)	22 (43.1)
Education level^c^				
Upper secondary school	110 (85.3)	34 (79.1)	29 (82.9)	47 (92.2)
Vocational school	19 (14.7)	9 (20.9)	6 (17.1)	4 (7.8)

Continuous variables are presented as means (SD) unless stated otherwise; *P* values were calculated with analysis of variance (ANOVA) or the Kruskal–Wallis test, as appropriate. Categorical variables are presented as *n* (%); *P* values were calculated with the chi-squared or Fisher’s exact test, as appropriate. All *P* values are non–significant, except for sex.

*Q1* 1st quartile, *Q3* 3rd quartile.

^a^Participants that attended study visit *n* = 117.

^b^*n* = 129.

^c^One participant out of school.

^d^*P* < 0.001, control group vs cow’s milk allergy (CMA) and CMA-refuted groups.

### Consumption of dairy products

There were no significant differences in median total dairy consumption between the CMA group, CMA-refuted group, and the controls (*P* 0.117) (Table 2). We divided the consumption of dairy into three categories – <100 g/d, 100–499 g/d, and ≥500 g/d – to examine this consumption as a categorical variable. Proportions were similar between groups (data not shown).

**Table 2:** Consumption of dairy products, vitamin D intake and supplement use.

	All (*n* = 127)	Cow’s milk allergy (*n* = 40)	Cow’s milk allergy refuted (*n* = 36)	Controls (*n* = 51)
Total dairy (g/d)	431 (137, 987)	449 (130, 1092)	566 (277, 1067)	235 (82, 871)
Liquid dairy (g/d)	421 (113, 987)	436 (111, 1082)	552 (277, 1042)	228 (64, 846)
Liquid dairy, proportion (<500 g/d)	72 (56.7)	22 (55.0)	17 (47.2)	33 (64.7)
Cow’s milk, drunk (ml/d)	100 (9, 400)	134 (24, 490)	172 (19, 430)	57 (1, 370)
Cheese (g/d)^a^	7.1 (1.4, 25.0)	10.0 (0.3. 25.0)	8.6 (3.6, 25.0)	3.6 (1.4, 10.0)
Vitamin D, total dietary intake (µg/d)	13.7 (6.3, 33.6)	16.7 (6.1, 32.9)	11.5 (6.6, 24.9)	14.8 (6.3, 44.1)
Vitamin D, intake from food (µg/d)	6.6 (4.6, 10.8)	7.0 (4.7, 11.9)	7.5 (4.3, 10.9)	6.0 (4.4, 8.6)
Vitamin D, intake from supplement (µg/d)^b^	20.0 (10.0, 42.9)	20.0 (13.6, 30.0)	20.0 (10.0, 50.0)	20.0 (9.3, 50.0)
Regular supplement use, proportion^c^				
Any	78 (60.0)	26 (60.5)	18 (50.0)	34 (66.7)
Vitamin D	74 (56.9)	25 (58.1)	17 (47.2)	32 (62.7)
Calcium	12 (9.2)	7 (16.3)^d^	0 (0)^d^	5 (9.8)

Continuous variables are presented as median (1st quartile and 3rd quartile); *P* values were calculated with the Kruskal–Wallis test. Categorical variables are presented as *n* (%); *P* values were calculated with the chi-squared or Fisher’s exact test, as appropriate. All *P* values are non–significant, except for use of regular calcium supplement. One patient with symptomatic cow’s milk allergy and one outlier with inconsistent questionnaire responses excluded unless stated otherwise.

^a^*n* = 129.

^b^*n* = 67.

^c^*n* = 130.

^d^*P* = 0.044, cow’s milk allergy (CMA) vs CMA refuted.

The median consumption of total dairy differed between males (896 g/d) and females (251 g/d) (*P* < 0.001), as well as between participants with (217 g/d) and without a special diet (470 g/d) (*P* = 0.002).

### Vitamin D concentration and intake from food and supplements

**Fig. 2:**
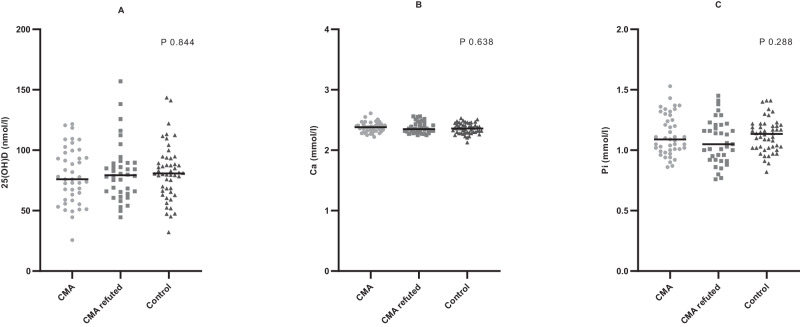
Vitamin D, calcium and phosphate levels in the three study groups; CMA, CMA refuted and control. All individual measures are plotted by group (circle = cow’s milk allergy (CMA) confirmed during infancy, CMA (*n* = 43); square = CMA refuted during infancy, CMA refuted (*n* = 38); triangle = control (*n* = 50)): (**A**) serum 25-hydroxyvitamin D concentration (nmol/l); (**B**) plasma calcium level (mmol/l); (**C**) plasma inorganic phosphate level (mmol/l). Lines represent median values. Groups were compared by the Kruskal–Wallis test; no differences between groups were significant.

We found no difference in median 25(OH)D concentrations between the CMA, CMA-refuted, and control groups (*P* = 0.844) (Fig. 2). Vitamin D insufficiency (25(OH)D < 50 nmol/l) was observed in 6.9% of all participants, and was similar across groups; 7% of the CMA group, 5.3% of the CMA-refuted group, and 8.0% of the control group (*P* = 0.914). The median 25(OH)D concentration did not differ between samples collected in May–June (73.0 nmol/l) and August–November (80.8 nmol/l) (*P* = 0.079). An analysis of median 25(OH)D concentrations between ethnicities (northern European (*n* = 111) vs other (*n* = 19)) showed no difference (*P* = 0.562) (data not shown).

The median dietary intake of vitamin D from food was 7.0, 7.5, and 6.0 µg/d for the CMA, CMA-refuted, and control groups, respectively (Table 2). Among all participants, 56.9% regularly consumed a vitamin D substitute; differences between groups were nonsignificant. The median dietary intake of vitamin D from supplements among those who reported regular supplementation was 20 µg/d in all groups (Table 2).

### Biochemical markers

We observed no differences in median calcium levels between the CMA (2.38 mmol/l), CMA-refuted (2.35 mmol/l), and control groups (2.36 mmol/l) or in median phosphate levels, which were 1.09 mmol/l, 1.05 mmol/l, and 1.14 mmol/l, respectively (Fig. 2). In the CMA group compared with the CMA-refuted group, consumption of a regular calcium substitute was significantly more frequent (Table 2).

### Healthy Eating Index and special diets

No differences were observed in median HEI between the CMA (30 points), CMA-refuted (32.5 points), and control groups (30 points) (*P* = 0.588). The prevalence of any special diet was 30%, 22%, and 45% in the CMA, CMA-refuted, and control groups, respectively (*P* = 0.071). Among all participants combined, lactose-free and vegetarian (consuming fish/eggs/dairy) were the most frequently reported special diets (Table 3).

**Table 3:** Prevalence of special diets among the three study groups.

	All (*n* = 130)	Cow’s milk allergy (*n* = 43)	Cow’s milk allergy refuted (*n* = 36)	Controls (*n* = 51)
Doctor-diagnosed lactose intolerance	8 (6.2)	2 (4.7)	1 (2.8)	5 (9.8)
Special diet				
Any	44 (33.8)	13 (30.2)	8 (22.2)	23 (45.1)
Low lactose or lactose free	19 (14.6)	5 (11.6)	3 (8.3)	11 (21.6)
Vegetarian; consumes milk, egg, and fish	15 (11.5)	2 (4.7)	5 (13.9)	8 (15.7)
Vegan	5 (3.8)	2 (4.7)	0 (0)	3 (5.9)
Gluten free or other	14 (10.8)	6 (14)	2 (5.6)	6 (11.8)

Variables are presented as *n* (%); *P* values were calculated with the chi-squared or Fisher’s exact test, as appropriate. All *P* values are non–significant.

## Discussion

In this rare clinical cohort of adolescents with a history of CMA and atopic eczema in infancy, we found no difference in dairy product consumption or vitamin D status compared with those with a negative CMA challenge in infancy and controls. Dietary intake of vitamin D was similar across groups, and no differences in HEI or prevalence of special diets were observed. The consumption of liquid dairy products was below the current national recommendation of a minimum of 500 g/d in 56.7% of all participants [27]. However, the majority (93.1%) were vitamin D sufficient.

Infants with CMA, at a mean age of 7 months, in comparison with healthy controls, have been shorter and thinner [28]. A reduction in height has also been described in young adults (median age 19.5 years) with symptomatic CMA compared with controls [29]. In children diagnosed with CMA at the mean age of 5.8 years, height data 1 year from diagnosis showed a significantly increased height SDS compared with at diagnosis among participants who had become tolerant [30]. This indicates that cessation of an elimination diet allows for catch-up growth. In our study, at the time of CMA diagnosis, prior to any elimination diet, infants were similar in weight and height compared to those with a negative DBPCFC. Similarly, in adolescence, our study found no difference between groups in height SDS or BMI-for-age.

In Finland, the reintroduction of milk into the diet, after CMA resolution, was studied from food records from 215 children that had received a special infant formula reimbursement and were followed up to 3 years of age [31]. At this age, 120 children had begun to use dairy products. The daily consumption of dairy products, after adherence to a milk elimination diet, was small as only 17% of children consumed three glasses of milk, and 23% used at least two slices of cheese, which are the national recommendations to ensure adequate intake of calcium [27]. In our study, the median intake of milk among adolescents with a history of CMA during infancy was less than one glass. However, this intake did not differ from that of nonallergic peers. After a negative cow’s milk oral food challenge, at the median age of 4.6 years (*n* = 41), introduction failure has been reported in 9.8% [32]. In our study, no milk-tolerant participants with a history of CMA during infancy or a negative DBPCFC during infancy had a failed cow’s milk reintroduction in adolescence. This suggests that the current clinical management of CMA successfully advances the discontinuation of unwarranted elimination diets. In addition, it may reflect the positive outcomes of the Finnish Allergy Program 2008–2018 [26]. After a two–year implementation only, an intervention based on the principles of the Finnish Allergy Program reduced allergy diet prevalence by 43% in a daycare setting [33, 34].

The majority of participants did not meet the national recommendation for liquid dairy product consumption. One plausible explanation is a notable increase, over the past two decades, in the consumption of plant-based beverages, which serve as a substitute for traditional bovine milk. This consumption has exponentially grown in Western Europe throughout the 21st century, while, simultaneously, the consumption of liquid dairy has consistently declined [35]. This increase in consumption of plant-based alternatives could have attenuated the differences between our study groups. Plant-based beverages in Finland are often fortified with vitamin D_2_, but compared to dairy products, the amount is less standardized. Notably, the updated Nordic Nutrition Recommendations 2023 for the intake of milk and dairy products is 350–500 g/d, whereas the previous national recommendation from 2014 was 500–600 g/d [27, 36]. While children aged 8 to 27 months on a milk-elimination diet ate more healthily compared with controls [12], our study on adolescents found no difference in diet healthiness as the HEI was similar across groups.

Interestingly, although only 57% of participants reported regular vitamin D supplementation, the prevalence of vitamin D insufficiency was only 6.9%. This is explained by the median vitamin D intake from food 6.6 µg/d, which is 66% of the adolescent daily recommended intake in Finland (10 µg/d) [27], and in line with previous findings among children [4]. Recommended daily supplementation is 7.5 µg/d all-year round for 2- to 17-year-olds [27]. Combining intake from food with intake from supplements raised the median intake among our participants to 13.7 µg/d, which is above the daily recommended intake. A previous study from Finland observed vitamin D insufficiency among 16% of 10-year-olds, concluding that, in these children, the vitamin D status was sufficient [7]. Our results support this finding, which is attributable to the national vitamin D fortification of liquid dairy products and fat spreads [3]. This study reaffirms the success of this fortification strategy in reducing vitamin D insufficiency. Our study found no difference in the 25(OH)D concentration between participants with a history of CMA during infancy compared with nonallergic peers. In contrast, in a previous report, children with a history of CMA (parental reported) showed a significantly lower mean 25(OH)D concentration (65.0 nmol/l) compared with children without CMA (74.0 nmol/l) [7]. An explanation could be that 19% of the children with CMA reported ongoing symptoms, whereas our analysis mostly included DBPCFC proven CMA participants who later had become milk tolerant. Another study found no differences in 25(OH)D levels between children aged 0.5 to 17 years with CMA, those with other food allergies, and controls [37]. In recent years, the heterogenous analytical performance of automated methods for measurement of 25(OH)D has been highlighted and liquid chromatography-tandem mass spectrometry remains the gold standard. However, automated immunoassays with <10% bias, such as ours, enable safe use in clinical practice [23].

The strength of our study is that CMA during infancy was either confirmed or refuted by DBPCFC, which for diagnosis of food allergy is the gold standard diagnostic test [38]. Also, children that develop tolerance are likely to do so by age 15 [8], thus, to study the reintroduction of dairy products, adolescence is an optimal period in life.

Limitations of this study include some of the control group traits, namely a high proportion of female participants and special diets. A report on special diets among 12- to 18-year-olds in Finland in 2013 found that the prevalence of special diets was 22.5% and that this prevalence was significantly higher in girls (28.4%) compared with boys (16.3%) [39]. The prevalence in our control group was 45%. Thus, the generalizability of our results may be compromised. Our data is also limited in terms of total milk intake from composite dishes. The applied FFQs were designed to evaluate the dietary intake of vitamin D and to calculate HEI; however, this limitation affects all groups equally and is unlikely to impact the results. Due to its cross-sectional nature, our study does not provide information on the timing of the reintroduction of cow’s milk or the span of the milk elimination diet in childhood. Even though no differences in vitamin D concentration or milk consumption were found in this population with a CMA history, other effects on health, such as bone strength or proneness to fractures, regardless of the cessation of an elimination diet, may persist. This warrants further research.

All participants in our cohort, with a history of CMA and developed milk tolerance, had successfully reintroduced dairy products into their diet. The nationwide fortification with vitamin D of liquid dairy and fat spreads has proven successful as almost all participants were vitamin D sufficient. Current management of children with CMA appears sufficient; additional monitoring of diet and vitamin D concentration, once milk tolerance has been attained, remains uncalled-for. Concerns regarding persisting with dietary restrictions that have surfaced in clinical contexts appear unwarranted.

## Data Availability

The data which was generated for and analyzed in this study is available from the corresponding author upon reasonable request.
